# Select Neuropeptides and their G-Protein Coupled Receptors in *Caenorhabditis Elegans* and *Drosophila Melanogaster*

**DOI:** 10.3389/fendo.2012.00093

**Published:** 2012-08-09

**Authors:** William G. Bendena, Jason Campbell, Lian Zara, Stephen S. Tobe, Ian D. Chin-Sang

**Affiliations:** ^1^Department of Biology, Queen’s UniversityKingston, ON, Canada; ^2^Cell and Systems Biology, University of TorontoON, Canada

**Keywords:** invertebrate neuropeptides, G-protein coupled receptor, insects, nematodes, *Caenorhabditis elegans*, *Drosophila melanogaster*

## Abstract

The G-protein coupled receptor (GPCR) family is comprised of seven transmembrane domain proteins and play important roles in nerve transmission, locomotion, proliferation and development, sensory perception, metabolism, and neuromodulation. GPCR research has been targeted by drug developers as a consequence of the wide variety of critical physiological functions regulated by this protein family. Neuropeptide GPCRs are the least characterized of the GPCR family as genetic systems to characterize their functions have lagged behind GPCR gene discovery. *Drosophila melanogaster* and *Caenorhabditis elegans* are genetic model organisms that have proved useful in characterizing neuropeptide GPCRs. The strength of a genetic approach leads to an appreciation of the behavioral plasticity that can result from subtle alterations in GPCRs or regulatory proteins in the pathways that GPCRs control. Many of these invertebrate neuropeptides, GPCRs, and signaling pathway components serve as models for mammalian counterparts as they have conserved sequences and function. This review provides an overview of the methods to match neuropeptides to their cognate receptor and a state of the art account of neuropeptide GPCRs that have been characterized in *D. melanogaster* and *C. elegans* and the behaviors that have been uncovered through genetic manipulation.

## Introduction

Animals respond to environmental cues through alteration of neural circuits that modify behavior and metabolism. The mechanism underlying the regulation of the neural circuit in response to a simple sensory cue is extremely complex and difficult to disentangle in mammals. The nematode *Caenorhabditis elegans* offers an excellent model organism to analyze neural circuit function. *C. elegans* is 1.3 mm in length and has several desirable features. The hermaphrodite has 959 somatic cells that form different organs and tissues including muscle, hypodermis, intestine, reproductive organs and glands, and the nervous system. The nervous system comprises 302 neurons whose identity and connections have been defined by morphology and ablation studies. *C. elegans* is also transparent such that *in vivo* fluorescent proteins and dyes can be used to visualize gene expression and fat metabolism in the living animal. The advantage over other systems is that alterations within a functional neuron can be studied in the context of the whole organism and rather than measuring changes in fluorescence, the actual behavioral response of the animal can be monitored directly. *Drosophila melanogaster* is more complex in terms of physical organization and behaviors. Some 90,000 neurons of the brain reflect a similar level of complexity as the different neural cell types in humans (Venken et al., 2011). *D. melanogaster*, like *C. elegans*, has a variety of genetic tools that allow for forward (mutagenic screens) and reverse (targeted gene disruption) genetics. The *Drosophila* UAS-Gal4 system (and derivatives there of) enable the use of specific promoters to target reporter genes, structural genes (for overexpression), or expression of ds RNA for selected gene inhibition (RNAi) to specific larval or adult tissues (Seroude, 2002). *D. melanogaster* and *C. elegans* have recently emerged as excellent paradigms for identifying the cellular mechanisms underlying human disease. Disease models are possible if the disease can be explained in molecular terms. Both of these non-mammalian genetic models have a strong record of uncovering disease-related genes that have orthologs in the human genome. These include the uncovering of genes involved in Alzheimer’s disease (Sundaram and Greenwald, 1993; De Strooper et al., 1999; Chakraborty et al., 2011), Parkinson’s (Feany and Bender, 2000), diabetes type II (Nakae et al., 2002), polyglutamine and other triplet repeat expansion diseases (Campesan et al., 2011), depression (Ranganathan et al., 2001; Pandey and Nichols, 2011), epilepsy, and schizophrenia (O’Kane, 2011).

*Drosophila melanogaster* and *C. elegans* produce and store most classical small molecule neurotransmitters in synaptic vesicles that cluster for release at pre-synaptic sites. These include acetylcholine, γ-aminobutyric acid (GABA), the biogenic amines octopamine, tyramine, dopamine, and serotonin (5-hydroxytryptamine) and glutamate (Bargmann, 2006) function in conjunction with neuropeptides that are produced as precursor proteins that are packed with processing enzymes and stored in large dense core vesicles in the trans-Golgi network. Release of neuropeptide-containing dense core vesicles is not restricted to nerve terminals and can occur at both axons and dendrites. In *C. elegans*, 113 neuropeptide genes have been identified that potentially produce 250 neuropeptides. These are classified into three groups: the insulin-like peptides (40 *ins* genes), the FMRFamide-related/like peptides (31 FaRP or *flp* genes), and the neuropeptide-like peptides (42 *nlp* genes; Nathoo et al., 2001). Two recent reviews summarize the synthesis, processing, and function of *C. elegans* neuropeptides (Husson et al., 2007; Li and Kim, 2008). In *D. melanogaster*, 119 neuropeptide genes have been predicted with 46 neuropeptide families characterized biochemically from 19 precursors (Clynen et al., 2010). In *C. elegans*, there are over 1100 G-protein coupled receptors (GPCRs) with approximately 100 thought to be specific for neuropeptides (Bargmann, 1998). *D. melanogaster* has approximately 160 GPCRs (far less than *C. elegans* with 44 exhibiting characteristics consistent with peptide ligand receptors (Hewes and Taghert, 2001). In both organisms, very few GPCRs have been matched with their respective neuropeptides and much less is known as to how each neuropeptide GPCR functions in neurotransmission or behavior. GPCRs can be separated structurally into several classes or subfamilies. The largest of these are the rhodopsin-like which are activated by small ligands and peptides. The secretin class of GPCRs have large extracellular domains that selectively bind glycoproteins. The metabotropic glutamate/pheromone GPCRs have domains that share sequence similarity with periplasmic binding proteins of bacteria involved in the transport of ions, amino acids, sugars, and peptides. The adhesion and frizzled class of GPCRs also have unique N-terminal binding domains with unique binding properties (Fredriksson et al., 2003; Krishnan et al., 2012). Given the diversity of GPCR types and varied functions this review focuses on some of the genetic and molecular techniques that have been used to specifically de-orphan neuropeptide GPCRs in *C. elegans* and *D. melanogaster* and decipher their role in regulating behavior and physiology.

## Matching Neuropeptides to Orphan Receptors

### Methodology

Only a limited number of reverse pharmacological approaches have been applied to match a peptide ligand to its receptor (i.e., de-orphanization) in *D. melanogaster* and *C. elegans*. All approaches are based on expression of the GPCR in a membrane system that will complete a signaling pathway that can be assayed. One of the more common assays used to de-orphan GPCRs is the GTPγS assay (Larsen et al., 2001). The GTPγS assay is one of the most sensitive assays for screening GPCRs and is widely used to characterize full and partial agonists and antagonists. In this assay, the GPCR of interest is expressed in mammalian cells such as Chinese hamster ovary (CHO) or human embryonic kidney (HEK293) cells. The plasma membrane replete with the recombinant GPCR of interest is purified and incubated with GDP and a potential neuropeptide ligand. A radiolabeled non-hydrolyzable GTP analog [^35^S] GTPγS, is then added. The premise of the assay is that if the neuropeptide has activated the receptor, the G-protein α-subunit exchanges GDP for GTP or in this case [^35^S]GTPγS which accumulates in the membrane and is easily measured. A second type of assay monitors cAMP levels. In this case, a receptor expressed in mammalian cells can be activated by adding a neuropeptide to the culture media. Upon activation, if exchange of GDP to GTP occurs using a Gα_s_ subunit, adenylate cyclase activity will be stimulated, converting ATP to cAMP. Conversely, if the GDP to GTP exchange occurs using a Gα_i_ subunit, adenylate cyclase is inhibited, and cAMP levels decline. In practice, a reporter construct that provides a promoter with multiple cAMP response elements controlling expression of the gene luciferase is co-transfected into cells with the receptor. Enhanced expression of luciferase occurs when cAMP increases. Luciferase catalyzes the oxidation of the firefly specific substrate, d-luciferin, to produce light which can be directly measured. If signaling occurs through Gα_i_, which depresses cAMP levels, cells can be treated with forskolin (which activates adenylate cyclase) prior to neuropeptide application. In this case, cAMP levels can be measured in cell extracts by incubation with a biotinylated-anti-cAMP antibody and a anti-cAMP antibody coupled to an acceptor bead. Streptavidin-coupled to a donor bead is then added to complex with biotin. Excitation of the donor bead with a laser (680 nm) produces singlet oxygen which can travel up to 200 nm and excite the cAMP – antibody bound acceptor bead in the complex. The acceptor bead then emits light which can be directly measured. Intracellular Ca^2+^ can also be used as a measure of GPCRs that couple through G_q_. G_q_ activates phospholipase Cβ which generates inositol triphosphate and diacylglycerol. Inositol triphosphate activates release of intracellular Ca^2+^ stores from the endoplasmic reticulum. Ca^2+^ can be measured by Ca^2+^ sensitive indicators such as Fluo-4. Alternatively, cells can be co-transfected with a gene that expresses apoaequorin. In the presence of the cofactor coelenterazine, a complex is formed that generates light proportional to the quantity of Ca^2+^. The relative simplicity of these assays has resulted in their widespread use in matching neuropeptides to their GPCRs, although the expression of *C. elegans* GPCRs in mammalian cells has encountered a number of pitfalls. For example, stable cell lines expressing some GPCRs cannot be generated because of toxicity problems. In addition, some GPCRs appear to be active only if cultured cells are incubated at 28°C rather than the normal 37°C (Harada et al., 1987; Geary et al., 1999; Kubiak et al., 2003a,b).

*Drosophila melanogaster* GPCRs have also been de-orphaned using a β-arrestin2-green fluorescent protein (GFP) translocation assay (Johnson et al., 2003). In this assay, following ligand-GPCR interaction in mammalian cells, β-arrestin2-GFP translocates from the cytoplasm to the cell membrane or receptor-bearing endosomes as part of termination of signaling (Barak et al., 1997).

G-protein coupled receptors of both *C. elegans* and *D. melanogaster* have also been expressed in *Xenopus laevis* oocytes along with a G-protein-gated inward rectifying potassium channel (GIRK; Harada et al., 1987). Gating results from release of the Gβγ subunits, which, upon receptor activation, then interact with GIRK. Measurement is through whole cell voltage-clamp recordings.

*Caenorhabditis elegans* GPCRs have been expressed in the pharynx of *C. elegans* by creating a transgenic animal with a GPCR construct that is under the control of a heat shock promoter. Action potentials are measured by placing a microelectrode into an exposed terminal pharyngeal bulb. For *C. elegans* neuropeptide receptor-1 (NPR-1), this method gave slightly different results than the *Xenopus* assay when the receptor was tested with several peptides (see below). Human somatostatin receptor and chemokine receptor 5 (CCR5) have been expressed in *C. elegans* nociceptive neurons ASH and ADL by transformation of the genes under the control of the *gpa-11* promoter. Transgenic animals showed an avoidance response to the cognate peptide placed between the worms and an attractant (Teng et al., 2006). This study has been extended to show that animals expressing CCR5 in nociceptive neurons will avoid *Escherichia coli* that expressed the natural ligand MIP-1α (Teng et al., 2008). As a cautionary note, this approach may only be applicable to non-modified peptides such as MIP-1α because *E. coli* does not have the enzymes necessary for some modifications, such as C-terminal amidation that some neuropeptides require for activity.

Despite the techniques outlined above, only a very small number of *C. elegans* and *D. melanogaster* receptors have been matched to their cognate ligand. At present, most families of known neuropeptides have been matched to receptors in *D. melanogaster* (Hewes and Taghert, 2001; Johnson et al., 2003; Clynen et al., 2010). The de-orphaning of *C. elegans* neuropeptide receptors has not been as rapid as in *D. melanogaster*. However, some of the *C. elegans* receptors that have been studied have provided better insights into components of the signal transduction pathways. Both model organisms though have advantages in that transgenic animals can be generated that overproduce neuropeptides or GPCRs and the availability of mutants that give rise to specific phenotypes that result from the suppression of neuropeptide and/or GPCR-linked functions.

## Comparing Function of Structurally Conserved Peptides and Receptors Identified in *Drosophila* and *Caenorhabditis*

Insect systems have proven invaluable in revealing primary peptide structures that define many neuropeptide families and for developing *in vitro* physiological assays that provide clues to *in vivo* functions. The signal transduction pathways for most neuropeptides though are only vaguely understood beyond their interaction with their cognate receptor. Genetic systems such as *D. melanogaster* and *C. elegans* are now extending our understanding of the steps between neuropeptide release to final physiological action. Many of these peptide-GPCR interactions lead to conserved functions. For example, allatostatin-like peptides appear to influence foraging behavior in *D. melanogaster* and *C. elegans*. These systems have also been instrumental in uncovering additional neuropeptide and neuropeptide GPCR functions.

### Neuropeptide F, NPY/NPF peptides, and receptors

In vertebrates, a 36 amino acid neuropeptide Y (NPY) functions as a neuromodulator to stimulate feeding behavior (Clark et al., 1984; Kalra, 1997). Roles of vertebrate NPY include suppression of responsiveness to adverse stimuli and in promotion of food search and acquisition under adverse conditions (Thorsell and Heilig, 2002). Destruction of NPY-expressing neurons in mice results in starvation of the animals (Pedrazzini, 2004). NPY is thought to work through a specific NPY receptor, to repress the activity of inhibitory neural circuits that then promotes feeding behavior (Klapstein and Colmers, 1993; Browning and Travagli, 2003).

In invertebrates, neuropeptide F is an ortholog of vertebrate NPY but differs in a C-terminal phenylalanine rather than tyrosine (Brown et al., 1999). *Drosophila* NPF (DromeNPF) is expressed in the brain and midgut of larvae and adults (Brown et al., 1999). A single receptor, Drome NPF receptor (DromeNPFR) has been identified through expression of the receptor in mammalian cells and binding assays (Garczynski et al., 2002; Table 1). In common with vertebrate NPY, DromeNPF, and its receptor have been associated with the control of social and feeding behaviors. DromeNPF levels are high in larvae, when they remain attracted to food, then fall to lower levels in subsequent developmental stages in which feeding is reduced. Overexpression of the DromeNPFR at later developmental stages encourages feeding behaviors that differ from wild type, whereas underexpression of DromeNPFR leads to a food aversion response in earlier larvae that would normally feed (Wu et al., 2003). Food-associated memory is promoted by starvation and inhibited by satiety in *Drosophila*. Stimulation of neurons that express DromeNPF mimics food deprivation and promotes memory performance in satiated flies. This memory performance requires the expression of the DromeNPFR in six dopaminergic neurons whereas blocking these neurons releases memory performance in satiated flies and suppresses memory performance in hungry flies. This suggests that dopamine and DromeNPF act together to coordinate memory performance, based on nutritional status (Krashes et al., 2009). DromeNPF also functions in fly-aversive responses to a variety of stressors. NPF appears to act antagonistically to insulin signaling systems in regulating aversive responses (Wu et al., 2005). In aversion responses, DromeNPF suppresses PAIN neurons through attenuation of transient receptor potential (TRP) channel-induced neuronal excitation (Xu et al., 2010). iDromeNPF signaling has been implicated in a wide range of behaviors and has recently been shown to have a modulatory effect on fly aggression. Drug-induced increases of 5-hydroxytryptamine (serotonin) amplify aggression amongst flies, whereas silencing of the serotonergic circuits leads to a lack of response following application of exogenous serotonin; however, the aggression response still exists. Silencing of the DromeNPF circuit leads to an increase in fly aggression, showing that serotonin and DromeNPF signaling systems act together to regulate fly aggression (Dierick and Greenspan, 2007). The DromeNPF signaling pathways also modulate acute ethanol sensitivity (Wen et al., 2005) and appear to have a male-specific function in courtship behavior. DromeNPF also has a possible role in circadian rhythms (Lee et al., 2006) and is potentially involved in the control of evening activity (Hermann et al., 2012). The signaling pathways regulating these later phenotypes are still poorly understood.

**Table 1:** **Receptor-ligand interaction affinities as measured in heterologous expression systems**.

Receptor gene	Peptide ligand	Peptide sequence	EC_50_	Reference
**C. elegans**				
Caeel C39E6.6 (NPR-1)	FLP-21	GLGPRPLRFa	**215V** 2.2–77 nM, **215F** 59–370 nM	Kubiak et al. (2003b)
**	FLP-21	GLGPRPLRFa	**215V** −108, **215F** −104	Rogers et al. (2003)
**	FLP-18-1	DFDGAMPGVLRFa	215V −9.3, 215F 0	
**	FLP-18-2	EMPGVLRFa	**215V** −32, **215F** 0	
**	FLP-18-3	SVPGVLRFa	**215V** −9.7, **215F** 0	
**	FLP-18-4	EIPGVLRFa	**215V** −8, **215F** 0	
**	FLP-18-5	SEVPGVLRFa	**215V** −6.8, **215F** 0	
**	FLP-18-6	DVPGVLRFa	**215V** −9.7, **215F** 0	
Caeel *npr-2* T05A1.1	Unknown			
Caeel *npr-3* C10C6.2	FLP-15-1	GGPQGPLRFa	162.4 nM	Kubiak et al. (2003a)
	FLP-15-2	RGPSGPLRFa	250.6 nM	
Caeel Y58G8A.4 (*npr-5)* Splice variants a and b	FLP-18-6	DVPGVLRFa	a: 32.3 nM; b: 45.9 nM	Rogers et al. (2003)
	FLP-18-3	(K) SVPGVLRFa	a: 28.3 nM, b: 48.3 nM	
	FLP-18-3 Truncated	SVPGVLRFa	a: 14.4 nM, b: 43.3 nM	
	FLP-18-5	SEVPGVLRFa	a: 25.9 nM, b: 58.4 nM	
	FLP-18-4	EIPGVLRFa	a:117.2 nM, b: 124 nM	
Caeel C26F1.6	FLP-7-2	TPMQRSSMVRFa	1.02 ± 0.24 μM	Mertens et al. (2005b)
	FLP-7 Truncated	SMVRFa	0.096 ± 0.016 μM	
	FLP-11-1	AMRNALVRFa	1.34 ± 0.41 μM	
	FLP-7-1	SPMQRSSMVRFa	Inactive	
	FLP-7-3	SPMDRKMVRFa	Inactive	
	FLP-7-4	SPMERSAMVRFa	Inactive	
Caeel Y59H11AL.1	FLP-7-3	SPMERSAMVRFa	<0.7 [28°C]–1.1 [37°C] μM	Mertens et al. (2006)
	FLP-7-2	TPMQRSSMVRFa	2.5 μM	
	FLP-7-1	SPMQRSSMVRFa	5.0 μM	
	FLP-7-4	SPMDRSKMVRFa	5.0 μM	
	FLP-1-8	KPNFMRYa	0.1 μM	
	FLP-9	KPSFVRFa	5.0 μM	
	FLP-11-1	AMRNALVRFa	0.75 μM	
	FLP-11-2	ASGGMRNALVRFa	2.5 μM	
	FLP-11-3	NGAPQPFVRFa	1.0 μM	
Caeel T19F4.1	FLP-2-1	SPREPIRFa	53.1 ± 7.7 nM	Mertens et al. (2005a)
	FLP-2-2	LRGEPIRFa^2^	54.4 ± 6.2 nM	
Caeel C46F4.1 (*egl-6)*	FLP-10	QPKARSGYIRFa	11 nM	Ringstad and Horvitz (2008)
	FLP-17-1	KSAFVRFa	28 nM	
	FLP-17-2	KSQYIRFa	1 nM	
Caeel C13B9.4 (*pdfr-1*)	PDF-1a	SNAELINGLIGMDLGKLSAVa	127.4 nM	Janssen et al. (2008b)
	PDF-1b	SNAELINGLLSMNLNKLSGAa	360 nM	
	PDF-2 NLP-37	NNAEVVNHILKNFGALDRLGDVa	33.8 nM	
Caeel;Y39A3B.5 (*ckr-2*)	NLP-12A	DYRPLQFa	56.7 nM	Janssen et al. (2008a)
	NLP-12B	DGYRPLQFa	14.7 nM	
**D. melanogaster**				
Drome NPFR CG1147	**DromeNPF CG10342**	SNSRPPRKNDVNTMADAYKFLQDLDTYYGDRARVRFa	65 nM*	Garczynski et al. (2002)
Drome sNPFR = NPRF76F CG7395	**Drome sNPF CG13968**	AQRSPSLRLRFa	5.1 × 10^−8^	Mertens et al. (2002)
		SPSLRLRFa	4.2 × 10^−8^	
		PQRLRWa	3.1 × 10^−8^	
		PMRLRWa	7.5 × 10^−8^	
DromeFR CG2114	Drome FMRF-1	DPKQDFMRFa	2.0 nM	Meeusen et al. (2002)
	Drome FMRF-2	TPAEDFMRFa	2.8 nM	
	Drome FMRF-3	SDNFMRFa	1.9 nM	
	Drome FMRF-4	SPKQDFMRFa	2.5 nM	
	Drome FMRF-5	PDNFMRFa	1.8 nM	
	*N. bullata*	APPQPSDNFIRFa	3.5 nM	
	*N. bullata*	pQPSQDFMRFa	2.0 nM	
Drome CG13229	Unknown			
Drome PDFR CG1758	Drome PDF CG6492	NSELINSLLSLPKNMNDAa	25 nM	Mertens et al. (2005c)
	Drome DH_31_	TVDFGLARGYSGTQEAKHRMGLAAANFAGGPa	218.6 nM	
Drome CCKLR	Drome DSK	FDDY(SO(3)H)GHLRFa	5.3 nM	Kubiak et al. (2002)

*Determined through a competition assay which displaced radioactive peptide from the receptor.

**Values represent alteration of current in response to neuropeptide application in the *Xenopus* assay.

The *C. elegans* GPCR (C39E.6.6 = Caeel *npr-1*) shares homology with the vertebrate NPY receptor family. Caeel *npr-1* is expressed in at least 20 neurons. Two natural alleles of NPR-1 that differ by a single amino acid at position 215, which likely affects G-protein signaling show two behavioral phenotypes termed “solitary” versus “social.” Animals, typified by the N2 Bristol strain used in most labs as a wild type strain, express Caeel *npr-1* with valine at position 215 (Caeel *npr-1* 215V). This Caeel *npr-1* allele results in animals with reduced locomotory activity on a bacterial lawn and they disperse over the lawn as solitary individuals. Animals such as the Hawaiian isolate CB4856, express Caeel *npr-1* with phenylalanine at position 215 (Caeel *npr-1* 215F). Caeel *npr-1* 215F animals display a “bordering” phenotype as they move rapidly on bacterial food to areas where the food is thickest, and burrow. These animals also show social behavior by interacting with each other to form clumps that can contain hundreds of animals (de Bono and Bargmann, 1998). Caeel NPR-1 acts as an inhibitor of social behavior as Caeel *npr-1* loss-of-function *(lf)* mutants display social behavior. This results in part from Caeel *npr-1* *(lf)* exhibiting enhanced attraction to low levels of the ascaroside pheromone (Macosko et al., 2009). The Caeel *npr-1* 215 Valine (V) allele is dominant to the Caeel *npr-1* 215 Phenylalanine (F) allele so that heterozygotes will display solitary behavior. Genetics and targeted expression studies have suggested that NPR-1 acts through neurons AQR, PQR, and URX that are exposed to body fluids (Coates and de Bono, 2002) to suppress aggregation and bordering by inhibiting the expression/activity of two α/β soluble guanylate cyclases GCY-35 and GCY-36 that are required to activate a cGMP-gated ion channel (TAX-2/TAX-4) encoded by the *tax-2* and *tax-4* genes (Cheung et al., 2004; Gray et al., 2005). Social animals may display aggregation and bordering activity as a means of avoiding high O_2_ levels (hyperoxia) on food. In solitary Caeel NPR-1 215V animals, food suppresses avoidance of hyperoxia by signaling through Caeel NPR-1 via GCY-35/GCY-36 and the TGF-β homolog DAF-7 (Cheung et al., 2005; Chang et al., 2006). On food, Caeel NPR-1 215V also promotes avoidance of high levels of CO_2_ whereas the Caeel NPR-1 215F-bearing animal only exhibits a weak avoidance to CO_2_. Indeed, an increase in CO_2_ leads to a burst of turning in wild type (N2) worms; however, the Caeel *npr-1 215F* strain does not respond. Up or downshifting of O_2_ has a dramatic effect on turning in Caeel *npr-1 215F*. The activity of Caeel NPR-1 may thus serve to integrate inputs from O_2_- and CO_2_-sensing pathways and generate an appropriate response with respect to availability of food (Bretscher et al., 2008; Chang and Bargmann, 2008; Hallem and Sternberg, 2008). The O_2_ and CO_2_-sensing pathways may control which peptides become involved in regulating Caeel NPR-1. A globin-like gene (*glb-5*) appears to cooperate with Caeel *npr-1* to mediate responses to O_2_ and CO_2_ concentrations. Expression of the globin-like gene (*glb-5*) in animals with a *lf* allele of Caeel *npr-1* showed suppressed aggregation behavior (McGrath et al., 2009).

Caeel NPR-1 has recently been shown to play a role in innate immunity, with Caeel *npr-1(lf)* animals showing an increased susceptibility to infection by the bacteria *Pseudomonas aeruginosa*. A similar initial signaling pathway may be used since one of the soluble guanylate cyclases (GCY-35) expressed in AQR, PQR, and URX neurons, and the cGMP-gated ion channel TAX-2/TAX-4 are required (Styer et al., 2008). Caeel *npr-1* has been implicated in hyperoxia avoidance in the presence of an exopolysaccharide matrix characteristic of mucoid bacteria. OSM-9 is part of the TRP Vanilloid (TRPV)-like ion channel that is within the ASH and ADL nociceptive neurons (Kapfhamer et al., 2008). The TRPV-like channel mutant (*osm-9*) mutant exhibited mucoid bacterial avoidance as a consequence of the lack of induction of the Caeel NPR-1 pathway. Worms that lack the TRPV-like channel and guanylate cyclase (*gcy-35*) showed restored Caeel NPR-1-dependent oxygen sensitivity and absence of pathogen avoidance exhibited by TRPV (*osm-9*) mutant (Reddy et al., 2011).

The TRPV-like channel appears to work with Caeel NPR-1 in several instances of behavioral adaptation/acute tolerance. For example, following exposure of wild type *C. elegans* to ethanol, intoxication can occur which is assayed by hyperexcitation followed by inhibition of locomotor activity and egg laying. Decreased intoxication as a result of acute tolerance is observed in Caeel NPR-1 215F animals which show a dramatic recovery to ethanol exposure relative to Caeel NPR-1 215V animals. Ethanol-induced clumping of animals was suppressed by the loss of the cGMP-gated ion channel (*tax-4*) and the TRPV-like channel (*osm-9*; de Bono et al., 2002). Caeel *npr-1* expression in RMG interneurons acts synergistically with TRPV-like channel (*osm-9*) in parallel pathways to regulate aversive behaviors at high temperature with *npr-1(lf)* animals show an increased threshold for heat avoidance (Glauser et al., 2011).

The initial study to de-orphan Caeel NPR-1 used expression of Caeel *npr-1* in a mammalian (CHO) cell line screened with 200 synthetic invertebrate peptide sequences. A single *Ascaris suum* neuropeptide AF9 (Cowden and Stretton, 1995) was identified as an activating ligand. AF9 is a FMRFamide-related peptide (FaRP) unrelated in sequence to NPY. This peptide sequence has been found in the *C. elegans* genome and is known as FMRFamide-like peptide-21 (FLP-21; Kubiak et al., 2003b). FLP-21 activation of Caeel NPR-1 results in inhibitory signaling through Gi/Go proteins and inhibition of cAMP production. In GTPγS binding assays, FLP-21 displayed higher activity with Caeel NPR-1 215V in comparison to Caeel NPR-1 215F (Kubiak et al., 2003b; Table 1). Higher FLP-21 activation of Caeel NPR-1 215V was also noted when the receptor was expressed in *Xenopus* oocytes and assayed for GIRK channel activation (Rogers et al., 2003; Table 1). This is consistent with the observation that social behavior is repressed in Caeel NPR-1 215V. In the *Xenopus* assay, in addition to FLP-21, six unique FaRPs encoded by *flp-18* were found to activate Caeel NPR-1 215 V but not Caeel NPR-1 215F (Table 1). Using a different assay system in which Caeel NPR-1 isoforms were expressed in the *C. elegans* pharynx, both isoforms were activated by the sole FLP-21 peptide and all FLP-18 peptides and signaling may occur through Gα_q_ (Rogers et al., 2003). The expression patterns of *flp-18* and *flp-21* have limited overlap. *flp-18* is expressed in neurons AVA, AIY, and RIG, motor neurons RIM and pharyngeal neurons M2 and M3. *flp-21* is expressed in sensory neurons ADL, ASE, and ASH, motor neuron MRA and pharyngeal neurons MC. M2 and M4. Deletion of *flp-21* has a limited effect on increasing aggregation and bordering in Caeel *npr-1* 215V animals but does enhance aggregation in Caeel *npr-1* 215F animals (Rogers et al., 2003). This supports a role for FLP-18 peptides acting in conjunction with FLP-21 to regulate behavior. However, this is not always the case as FLP-21 does not appear to act in acute ethanol tolerance, suggesting that FLP-18 may be the active ligand. FLP-21 may act solely with Caeel NPR-1 in adaptation to heat avoidance (Glauser et al., 2011).

C53C7.1 is another *C. elegans* receptor related to the *Drosophila* neuropeptide F-like receptor. Two isoforms of C53C7.1 are generated by alternative splicing. A single patent report identifies a different FMRFamide-like peptide encoded by the *flp-3* gene as the ligand for C53C7 (Lowery et al., 2003).

### Short NPF and sNPF receptors

The *D. melanogaster* gene for short NPF (sNPF) encodes a precursor polypeptide that, upon processing, would release two C-terminal RLRFamides (sNPF-1, sNPF-2) and two RLRWamides (sNPF3, sNPF4; Hewes and Taghert, 2001; Vanden Broeck, 2001). Several thousand neurons in the CNS of *D. melanogaster* express sNPFs suggesting an array of potential functions for these neuropeptides (Nassel et al., 2008). A single neuropeptide GPCR (NPRF76F, CG7395, sNPRF1) is activated by all four sNPFs (Mertens et al., 2002; Table 1). Many sNPF-expressing neurons also co-express classic neurotransmitters suggesting that sNPF could act as a co-transmitter or neuromodulator (Nassel et al., 2008). sNPFs are also produced in the hypocerebral ganglion and in the anterior and middle midgut of *D. melanogaster* although the function of sNPF at these sites is unknown (Veenstra et al., 2008). In *D. melanogaster*, sNPF and sNPFR1 regulate body growth and metabolism by activating extracellular signal-related kinases (ERK) that mediate insulin-like peptide signaling (Lee et al., 2004). This response appears to show evolutionary conservation as mammalian NPY also mediates growth, metabolism, and lifespan through ERK-mediated insulin signaling. Olfactory receptor neurons also express sNPF (Carlsson et al., 2010) and this expression is responsible for starvation-dependent enhancement of food searching behavior. Starvation results in an insulin signal that functions via the phosphoinositide 3-kinase pathway to upregulate sNPFR1 expression in odorant receptor neurons which in turn sensitizes select sensory neurons to promote food search behavior (Root et al., 2011).

In *C. elegans*, the sequence of NPR-2 (T05A1.1) shares 26% amino acid sequence identity with the *D. melanogaster* sNPFR1. Three isoforms of NPR-2, one of 430 aa and two of 387 aa that differ at their amino-terminus, could potentially be generated by alternative splicing. RNAi experiments have demonstrated that knockdown of NPR-2 expression leads to a reduction in locomotion (Keating et al., 2003). Reduction in NPR-2 expression is also associated with an increase in accumulation of intestinal lipid (Cohen et al., 2009). The endogenous ligand is unknown. FLP-18 and FLP-21 were tested in the *Xenopus* oocyte assay and were unable to activate NPR-2 (Cohen et al., 2009).

*Caenorhabditis elegans npr*-3 (C10C6.2) is currently thought to specify only one GPCR of 376 aa that is expressed in the nerve cord and in excitatory and inhibitory motorneurons in the region of the nerve cord. The NPR-3 sequence, like NPR-2 is most related to the *D. melanogaster* sNPFR1 sharing 30% amino acid sequence identity. In *C. elegans*, the functions of this GPCR are still being established. In one RNAi screen, a knockdown of NPR-3 led to abnormal locomotion, with animals exhibiting sluggish behavior and a flat locomotory path as body bends were reduced (Keating et al., 2003). A second RNAi screen found that reduction of NPR-3 in a *lin-35* background (loss of *lin-35* results in enhanced RNAi) led to reduced brood size and protruding vulvas (Ceron et al., 2007). Neuropeptides specified by *flp-15* appeared to be the only peptides that could activate NPR-3 in a GTPγS assay with membranes prepared from *npr-3* transiently transfected CHO cells. In this assay, FLP-15-2 was a more potent activator than FLP-15-1 (Table 1). This interaction appears to be specific since a peptide such as FLP-21 (Table 1), which shares the carboxyl-terminal GPLRFamide, was inactive. NPR-3 appears to require a distinct conformation as activity was only observed when transiently NPR-3 transfected cells were incubated at 28°C. Membranes prepared from cells incubated at 37°C were inactive. Attempts to make a stably transformed NPR-3 cell line with CHO, HEK293, or COS7 cells were unsuccessful and resulted in cell death. In a transient expression assay; NPR-3 appears to activate via pertussis toxin-sensitive Gi/Go coupled signaling pathways which suggests that NPR-3 action is inhibitory (Kubiak et al., 2003a).

A second potential sNPF-like GPCR in *C. elegans* is Y58G8A.4 (NPR-5). NPR-5 RNA is spliced to generate two receptor isoforms of 397 aa (NPR-5a) and 433 aa (NPR-5b) that differ in sequence at the carboxyl-terminus. NPR-5 is most similar (31% amino acid sequence identity) to the *D. melanogaster* receptor CG7395 that encodes a NPF-like GPCR; that binds sNPF (Mertens et al., 2002). Both isoforms of NPR-5 were assayed for activation with 150 synthetic peptides in a transient expression system in CHO cells. The most potent activators in a Ca^2+^ mobilization assay were peptides derived from the *flp-18* gene. FLP-18 peptides showed activation with EC_50_ values in the nM range, with most having similar potencies using either NPR-5a or b (Table 1). The least active peptide was the longest FLP-18-1 which is also the least active when assayed with the NPF-like receptor NPR-1 (Rogers et al., 2003). FLP-18-1 has been isolated as a processed peptide with the first three amino-terminal amino acids removed which may result in a more potent form of the peptide (Clynen et al., 2009). The sole FLP-21 peptide, that is the cognate ligand for NPR-1, was found to activate both forms of NPR-5 but with far less potency (Kubiak et al., 2008). This is not surprising since FLP-18 peptides have been shown to activate NPR-1 in oocyte expression assays as well as in a *C. elegans* pharyngeal expression assay (Rogers et al., 2003). It is unclear whether the FLP-18 and FLP-21 peptides work together. The two isoforms of NPR-5 may activate multiple signal transduction pathways as contributions from G_q_, G_s_, and G_i_ were observed (Kubiak et al., 2008). Deletion mutants of *flp-18* display no measurable phenotype.

### FMRFamides and FMRFamide-related receptors

In vertebrate systems, neuropeptides with C-terminal sequence FMRFamide and FaRPs function in regulation of muscle contraction, feeding behavior, and learning and memory (Panula et al., 1996).

In *D. melanogaster*, FMRFamides are expressed from a single gene that encodes a precursor specifying 8 FMRFamide peptides. Five copies of the peptide Drome FMRF-1 would be released from the precursor (Table 1; Schneider et al., 1993). *In vitro* assays have established that FMRFamides function as modulators of muscle contraction, including in larval heart muscle; crop, foregut, and muscle of the body wall (Nichols et al., 2002; Nichols, 2003). The *D. melanogaster* FMRFamide GPCR (CG2114; Drome FR) is expressed in most larval and adult tissues. Drome FR was de-orphaned in two independent studies. In an aequorin bioluminescence assay, Drome FMRFamides 1–5 (numbered as unique FMRFamide-terminating peptide sequences from amino to the carboxyl-terminus of the precursor) were found to elicit a calcium response in a dose-dependent manner in CHO expressing (Table 1). *Neobellieria bullata* FMRFamide peptides were found to be active with the Drome FR with comparable potencies to native Drome FMRFamides (Table 1; Meeusen et al., 2002). Drome FMRFamide-5 was the most potent ligand in both studies (Cazzamali and Grimmelikhuijzen, 2002; Meeusen et al., 2002). Both studies found that the Drome FR can be activated by non-FMRFamide peptides such as Drome sNPF-1 and Drome myosuppressin; however, these peptides require much higher concentrations to elicit a response. Despite the high concentration required, FMRFamides were recently shown to act post-synaptically, inducing slow larval body wall contractions and increased tonus of the body wall muscles. The latter action of Drome FMRFamide requires activation of Drome FR and the Drome myosuppressin GPCR as well as an influx of calcium through L-type calcium channels (Klose et al., 2010). A role in reproduction had been suggested for Drome FR (Meeusen et al., 2002) as it is related in sequence to a sex peptide receptor (CG16752); however, the Drome FR could not replace the sex peptide receptor in *in vitro* expression assays (Yapici et al., 2008). Drome CG2114 shares 16–20% amino acid identity with *C. elegans* GPCRs F21C10.9 and C26F1.6. Following knockdown of the expression of the *C. elegans* receptor C26F1.6 by RNAi, a hyperactive egg laying phenotype is observed suggesting that this GPCR functions in control of egg production (Keating et al., 2003). Using expression of Caeel C26F1.6 in mammalian cells, only two neuropeptide sequences elicited a dose-dependent response Peptide FLP-7-2 which is found as two copies within the *flp-7* gene-encoded precursor was the most active followed by FLP-11-1 which is one of four peptides specified by the *flp-11* gene. Related peptides FLP-7-1, FLP-7-3, and FLP-7-4 processed from the FLP-7 precursor were inactive (Mertens et al., 2004, 2005b; Table 1). The FLP-7-2 peptide is likely cleaved at the arginine at the fifth position from the amino-terminus, as truncating the peptide to the terminal 5 amino acids was more active in receptor activation than the predicted full-length peptide (Mertens et al., 2005b). If processing does occur, all peptides from the FLP-7 precursor could be active peptides for receptor Caeel C26F1.6. Alternatively, the unique amino-terminal sequences may be required for targeting.

Caeel Y59H11AL.1 is a FaRP receptor that is related to the invertebrate tachykinin/mammalian neurokinin family of receptors. Caeel Y59H11AL.1 is most closely related to the *Drosophila* NPY-like receptor (CG5811, DromeNepYr) which is a tachykinin family member. Drome NepYr has not been assigned a functional role. However, the Caeel Y59H11AL.1 receptor appears to play a role in growth and reproduction as knockdown of Caeel Y59H11AL.1 gene expression results in small animals with a reduced brood size (Ceron et al., 2007). Expression of the Caeel Y59H11AL.1 gene results in two potential RNA splice variants that lead to two receptors of 427 aa and 434 aa. The two receptors differ by alteration of peptide sequence at the carboxyl-terminal region of the receptor. Of 68 neuropeptides tested against Caeel Y59H11AL.1 expressed in mammalian cells, the Caeel *flp-7* gene-encoded peptide FLP-7-3 was the most potent peptide (Table 1; Mertens et al., 2006). Three other peptides processed from the Caeel FLP-7 precursor, FLP-7-1, FLP-7-2, and FLP-7-4 were less active. Peptide FLP-7-4 appears to be the only Caeel FLP-7 precursor-derived peptide that uniquely activates Caeel Y59H11AL.1, as the others activate Caeel C26F1.6 as well. This result is surprising since the two receptors share limited sequence identity. Other peptides that showed weak activation of Caeel Y59H11AL.1 were Caeel FLP-1-8, FLP-9, and FLP-11-1-3 (Table 1; Mertens et al., 2006). Activation by multiple related peptides suggests a functional redundancy in peptide binding or possibly a less selective requirement of the receptor to respond to a variety of signals.

Another FaRP receptor in *C. elegans* is T19F4.1. RNA splice variants give rise to two receptors of 402 aa (Caeel T19F4.1a) and 432 aa (Caeel T19F4.1b). The difference between the receptors resides with the intracellular carboxy-terminus. After transient expression of each receptor in mammalian cells, two peptides, FLP-2-1 and FLP-2-2 derived from the Caeel *flp-2* gene precursor were found to activate in a dose-dependent manner either Caeel T19F4.1a or Caeel T19F4.1b (Table 1). A stable transformed mammalian cell line expressing Caeel T19F4.1b showed far less activation with FLP-2-1 and was less responsive when challenged with FLP-2-2 (Mertens et al., 2005a). The reasons for this are unclear. In a genome-wide RNAi screen, knockdown of the Caeel *flp-2* gene resulted in lethality in the embryo or larval stages or resulted in postembryonic growth defects (Simmer et al., 2003). No visible phenotypes have been identified in a Caeel FLP-2 receptor knockdown that would affect both splice variants.

Caeel C46F4.1 GPCR was found to be involved in an egg laying-defective phenotype (egl) in *C. elegans*. The most related receptor in *D. melanogaster* is Drome CG13229; however, no ligand or function has been ascribed to this unnamed fly receptor. Flyatlas lists low expression in the *D. melanogaster* nervous system. Caeel C46F4.1 is equivalent to *egl-6* (Ringstad and Horvitz, 2008) and two receptor isoforms that differ at the amino-terminus are produced by alternative splicing and alternate start sites. Caeel *egl-6* is predominately expressed in HSN motor neurons that innervate vulval muscles and glia-like cells located in the head region. Weaker expression was also noted in DVA tail interneurons. Expression was sometimes seen in lateral interneurons SDQL and SDQR (Ringstad and Horvitz, 2008). A gain-of-function mutant (n592*gf*) that results from a single amino acid change, Alanine 135 to Threonine 135, in the third transmembrane domain enhances EGL-6 activity. The result of this receptor activation is an egg laying-defective phenotype. Thus, EGL-6 normally transduces signals that confer inhibitory activity on the HSN motor neurons. This activity is dependent, in part, on G_o_ signaling. Transgenic overexpression of Caeel *flp* and other neuropeptide genes in both wild type and animals that carried an *egl-*6 deletion suggested that the ligands for EGL-6 were dependent on Caeel *flp-10* and Caeel *flp-17* genes. This was further supported by the demonstration that a Caeel *flp-10* deletion mutant suppressed the egg laying defect in the gain-of-function mutant and suppression was further enhanced by deletion of the Caeel *flp-17* gene. Peptides FLP-10 and two unique peptide sequences FLP-17-1 and 2, proved to be potent activators of EGL-6 when expressed in *X. laevis* oocytes. A GIRK channel assay, used to monitor expression, demonstrated that all peptides were potent activators, with EC_50_ values in the nM range (Table 1). Expression of Caeel *flp-*17 is confined to anterior BAG sensory neurons and this expression is necessary for EGL-6 function in egg laying. Expression of Caeel *flp-10* occurs in numerous neurons ASIL, ASIR, DVB, PVCL, PVCR, PVR as well as in non-neuronal tissues including head mesodermal cells, vulval tissue, uterine cells, and spermathecae. Only non-neuronal expression of Caeel *flp-10* appears to be important in EGL-6 action on egg laying (Ringstad and Horvitz, 2008).

### Pigment dispersing factor and receptor

Pigment dispersing hormone is a light adapting hormone originally identified as responsible for daily rhythms of color change in Crustacea (Meelkop et al., 2011). Similar peptides known as pigment dispersing factors (PDFs) have been identified in arthropods and they are required for normal circadian control of locomotion. In *D. melanogaster*, the PDF receptor (DromePDFR; CG13758) null mutant does not show any apparent morphological defects. Drome PDFR immunostaining revealed that DromePDFR was localized in 13 neurons in each hemisphere of the adult brain, 4 I-LNV neurons, one of the six LNd neurons, seven neurons in the DN1 area, and one neuron in the DN3 area. In a 12-h Light/Dark cycle, the receptor null mutants started morning activity later than wild type and began evening activity earlier than wild type. Placing animals in 24 h dark conditions for 8 days resulted in a loss of rhythmic activity. This behavior is seen in *Drosophila* PDF mutants, suggesting that DromePDF and DromePDFR may be part of a pathway that controls rhythmic circadian behavior. DromePDFR can be activated with Drome PDF, pituitary adenylate cyclase activating polypeptide (PACAP), or calcitonin-like peptides (Drome DH_31_; Mertens et al., 2005c).

A search of the *C. elegans* database for a PDF receptor (PDFR) ortholog using the *Drosophila* PDFR (CG13758) as a query sequence (Renn et al., 1999) resulted in the identification of the gene Caeel C13B9.4 as coding for a Caeel PDFR. Transcription and alternate splicing of gene Caeel C13B9.4 mRNA gives rise to six mRNAs (Wormbase). Two mRNAs that differ by variable length of the 5′ untranslated region specifies one of three related receptors. These three receptor isoforms differ at the amino-terminal region of the proteins, generating receptors of 543 aa (Caeel PDFR-1a); 536 aa (CaeelPDRF-1b), and 541 aa (Caeel PDRF-1c). Caeel C13B9.4 promoter-driven reporter expression showed that expression of this receptor is extensive. Expression was observed in chemosensory neurons PHA and PHB, mechanosensory neurons PLM, ALM, FLP, OLQD, and OLQV, the ring motor neuron RMED, the I1 pharyngeal interneuron pair, and a single sensory neuron R3 in the male tail. In non-neuronal tissue, expression was found in 95 body wall muscles and two vulval cells (Janssen et al., 2008b). This localization is similar to the localization of PDF-like neuropeptides which are found in neurons involved in chemosensation, mechanosensation, oxygen sensing, and locomotion (Janssen et al., 2008b). Expression of each of the three receptor isoforms in stably transformed CHO cells demonstrated that only three of 156 synthetic *C. elegans* peptides were active in a calcium bioluminescence assay. These included Caeel PDF-1a, PDF-1b, and PDF-2 = NLP-37 (Table 1). These three peptides were most active with Caeel PDFR-1b but showed reduced activity with Caeel PDFR-1a. Caeel PDFR-1c was inactive in this assay. In a cAMP activation assay in transiently transfected HEK293 cells, all three receptor isoforms were activated by Caeel PDF-1a, Caeel PDF-1b, and Caeel PDF-2, which suggests signaling through Gα_s_. In this assay, Caeel-PDFR-1b was the most active and responded particularly to Caeel PDF-2. Caeel PDFR-1c was the least active isoform. Cells expressing Caeel PDFR-1 responded to all three peptides in a dose-dependent manner, using the ability to inhibit forskolin-induced cAMP formation, which suggests that Caeel PDFR-1c may participate in coupling with additional G-proteins such as Gα_i/o_.

A Caeel *pdf-1* *(lf)* results in a decrease in the speed of movement of worms, an increase in the reversal frequency and an increase in the frequency of directional change. The net result is that the distance that animals travel in the forward direction is dramatically reduced. These locomotor defects are not seen in animals in which Caeel *pdf-1* is over-expressed. In contrast, overexpression of Caeel *pdf-2* results in a phenotype equivalent to the Caeel *pdf-1(lf)*. Overexpression of Caeel *pdfr-1* (expressing all 3 isoforms) results in animals that show a dramatic increase in reversal frequency but lack changes in speed of movement or directional change. The current model is that Caeel PDF-1 peptides activate Caeel PDFR-1 to stimulate forward movement and/or inhibit backward movement and this effect is counter-balanced by Caeel PDF-2 acting on Caeel PDFR-1 to inhibit forward movement and/or stimulate backward movement (Janssen et al., 2008b). *D. melanogaster* clock genes have counterparts in *C. elegans*. Null alleles of *C. elegans* clock genes reduced mRNA levels of Caeel *pdf-1a*, *pdf-1b*, and *pdf-2* which implicates Caeel PDF-1 and 2 activity as dependent on the clock genes. Caeel *pdf-1* appears to work independently of Caeel *pdf-2* as the level of one does not affect the other (Janssen et al., 2009).

### Cholecystokinin and its receptor

Cholecystokinin (CK) is known in vertebrates as a regulator of food intake as it functions to stimulate smooth muscle contraction which, in vertebrates, includes intestinal and gall bladder contractions. CK also stimulates the secretion of digestive enzymes such as α-amylase (Dufresne et al., 2006). The *D. melanogaster* CK-like receptor (Drome CCKLR) was identified based on homology to mammalian CK receptors (CKR) and was found in mammalian expression assays to bind to a sulfated FMRFamide-like peptide, drosulfakinin (Drome DSK). The sulfated form of Drome DSK is necessary to achieve specific interaction with EC50 values in the nM range (Kubiak et al., 2002). Analysis of loss-of-function mutations in either Drome CCKLR or Drome DSK results in neuromuscular junction undergrowth suggesting that both GPCR and ligand are required pre-synaptically to promote neuromuscular junction growth. Genetically, Drome CCKLR and Drome DSK were found to function upstream of Gαs which in turn regulates a cAMP-dependent protein kinase which then acts on a transcriptional regulatory protein CREB2 which is the primary effector of the pathway (Chen and Ganetzky, 2012). In *C. elegans*, CaeelY39A3B.5 shares 67% similarity with mammalian CKR (CCK2R) and 64% with sulfakinin receptors (DK-R1; Johnsen, 1998; Janssen et al., 2008a). Through computer predicted alternate splicing, Caeel Y39A3B.5 produces four isoforms of 582 aa (Y39A3B.5a), 552 aa (Y39A3B.5b), 471 aa (Y39A3B.5c), and 617 aa (Y39A3B.5; Wormbase). Additional isoforms may exist as two further isoforms were identified as a result of sequencing DNA generated experimentally by reverse-transcriptase PCR. Both contained the first eight exons of isoform c but then differed, as one contained the last two exons of isoform b (Y39A3B.5c/b = Caeel CKR-2a) and the second the last four exons of isoform *d* (Y39A3B.5c/d = Caeel CKR-2b). These two receptors were de-orphaned by transient expression in CHO cell lines, using a calcium bioluminescence assay. Caeel NLP-12a and Caeel NLP-12b were the only peptides tested that activated Caeel CKRs in a dose-dependent manner (Table 1). The most potent ligand for Caeel CKR-2a was Caeel NLP-12b whereas NLP-12a showed a higher potency than NLP-12b with CKR-2b. NLP-12 is localized to a tail interneuron DVA and to processes from DVA that extend around the nerve ring. Expression was also observed in all six coelomocytes. In common with vertebrates, Caeel NLP-12 can regulate digestion since Caeel *ckr-2(lf)* have decreased intestinal α-amylase and both Caeel *ckr-2(lf)* and Caeel *nlp-12(lf)* animals gain fat even though there is no difference in pharyngeal pumping rate or defecation rate. Caeel *ckr-2* and its ligand, Caeel *nlp-12*, may also be involved in a mechanosensory feedback loop that couples muscle contraction to changes in pre-synaptic ACh release (Hu et al., 2011).

### Allatostatin-like peptides and receptors

Mammalian galanin is a neuropeptide that regulates numerous physiological processes including neurotransmission, nociception, feeding and metabolism, energy, and osmotic homeostasis as well as learning and memory (Lang et al., 2007). Insect allatostatins (ASTs) have a carboxyl-terminal sequence Y (Xaa) FGL-amide and have multiple functions that include inhibition of juvenile hormone biosynthesis (Bendena et al., 1999; Tobe and Bendena, 2012) inhibition of muscle contraction, regulation of digestive enzymes, and neuromodulation (Tobe and Bendena, 2012). In *Drosophila* Drome FGL-amide ASTs do not inhibit juvenile hormone biosynthesis. RNAi reduction in Drome AST or Drome ASTR transcripts results in reduced locomotory behavior in the presence of food. Locomotion is normal in the absence of food. Reduction in Drome AST and Drome ASTR is correlated with decreased *for* transcript levels which encodes cGMP-dependent protein kinase. A reduction in the *for* transcript is known to be associated with a naturally occurring allelic variation that creates a sitter phenotype in contrast to the rover phenotype which is caused by a for allele associated with increased for activity (Wang et al., 2012). In *C. elegans* the gene Caeel *npr-9* expresses a single GPCR isoform of 444 aa that shares 33 and 37% amino acid sequence identity with mammalian galanin receptor 2 and the Drome allatostatin receptor (Drome ASTR), respectively. Promoter-driven reporter expression suggests that Caeel *npr-9* is transcribed exclusively in interneuron AIB. Caeel NPR-9 appears to function as an inhibitor of local search behavior in the presence of a food stimulus. In the absence of food. Caeel *npr-*9 *(lf)* mutants display locomotory activity that is identical to wild type animals. Caeel *npr-9* *(lf)* mutants behave as if AIB is stimulated (increased pivoting and local search). Caeel *npr-9(lf)* animals also accumulate fat at an accelerated rate relative to wild type and thus again resemble galanin/allatostatin neuropeptides that affect metabolism. This contrasts with Caeel npr*-9(gf)* animals (overexpression of Caeel NPR-9) which display enhanced forward locomotion that mimic the phenotype displayed by AIB laser ablation or a mutation in the glutamate receptor-1 (Bendena et al., 2008). Caeel *npr-9(gf)* animals travel long distances off food, presumably as a result of overriding dopamine, and glutamate signals that evoke “area restricted search” behavior in wild type animals. Area restricted search is characterized by frequent reversals and sharp omega-turns that function to maximize the time spent on an abundant food source (Hills et al., 2004). The ligands for Caeel NPR-9 have not yet been identified. Two genes, Caeel *nlp-5* and Caeel *nlp-6*, specify peptides that resemble ASTs. Caeel *nlp-5* and Caeel *nlp-6* specify peptides with carboxyl-terminal MGLamide and MGFamide, respectively. Caeel *nlp-6* encodes a peptide with carboxy-terminal FGFamide. A mutation in Caeel *nlp-5* has been reported to result in animals with altered locomotory behavior on food (Bargmann, Wormbase), which appears to be similar to behaviors exhibited by Caeel *npr-9(lf)* animals.

## Perspectives

High throughput neuropeptide projects are expected to facilitate de-orphanization of all of the predicted *D. melanogaster* and *C. elegans* neuropeptide receptors. These neuropeptides and their receptors will serve as starting points to understand the functional significance of these signaling events. Both organisms serve as genetic models not only for matching GPCRs with their respective neuropeptide ligand but offer a means of uncovering signal transduction pathways that lead to novel behaviors. Genetic modifier screens and genome-wide RNAi screens will certainly identify many of the neuropeptide signaling components. *C. elegans* transgenic studies will allow the manipulation of neuropeptide receptor signaling at the level of a single cell or tissue within an entire organism. As many of these receptors have counterparts in mammals, it will not be surprising to find similar signaling pathways conserved throughout evolution.

## Conflict of Interest Statement

The authors declare that the research was conducted in the absence of any commercial or financial relationships that could be construed as a potential conflict of interest.
